# Speciation and milk adulteration analysis by rapid ambient liquid MALDI mass spectrometry profiling using machine learning

**DOI:** 10.1038/s41598-021-82846-5

**Published:** 2021-02-08

**Authors:** Cristian Piras, Oliver J. Hale, Christopher K. Reynolds, A. K. Jones, Nick Taylor, Michael Morris, Rainer Cramer

**Affiliations:** 1 Department of Chemistry, University of Reading, Whiteknights, Reading, RG6 6DX UK; 2 Department of Health Sciences, “Magna Græcia University” of Catanzaro, Campus Universitario “Salvatore Venuta” Viale Europa, 88100 Catanzaro, Italy; 3 School of Agriculture, Policy and Development, University of Reading, Whiteknights, Reading, RG6 6EU UK; 4 Veterinary Epidemiology and Economics Research Unit (VEERU) & PAN Livestock Services Ltd, School of Agriculture, Policy and Development, University of Reading, Whiteknights, Reading, RG6 6EU UK; 5 Waters Corporation, Stamford Avenue, Wilmslow, SK9 4AX UK; 6 School of Biosciences, University of Birmingham, Edgbaston, Birmingham, B15 2TT UK

**Keywords:** Proteomics, Biological techniques, Mass spectrometry, Biochemistry, Lipids, Proteins

## Abstract

Growing interest in food quality and traceability by regulators as well as consumers demands advances in more rapid, versatile and cost-effective analytical methods. Milk, as most food matrices, is a heterogeneous mixture composed of metabolites, lipids and proteins. One of the major challenges is to have simultaneous, quantitative detection (profiling) of this panel of biomolecules to gather valuable information for assessing food quality, traceability and safety. Here, for milk analysis, atmospheric pressure matrix-assisted laser desorption/ionization employing homogenous liquid sample droplets was used on a Q-TOF mass analyzer. This method has the capability to produce multiply charged proteinaceous ions as well as highly informative profiles of singly charged lipids/metabolites. In two examples, this method is coupled with user-friendly machine-learning software. First, rapid speciation of milk (cow, goat, sheep and camel) is demonstrated with 100% classification accuracy. Second, the detection of cow milk as adulterant in goat milk is shown at concentrations as low as 5% with 92.5% sensitivity and 94.5% specificity.

## Introduction

Growing concerns about physical well-being and public health have increased the demand for healthier foods with different nutraceutical properties^1^. For this reason, consumers look for alternatives to cow milk such as goat and camel milk. On the other hand, the commercial value of these alternative products is often higher, and therefore more susceptible to adulteration for fraudulent economic gain.

In modern food production and retail it is important to have rapid and cost-effective analytical methods available for assuring food quality and safety. One of the most frequent adulterations in the field of dairy products is related to the fraudulent addition of cow milk to milk of other species^2^. This represents a matter of food quality and authenticity but is also a matter of safety for consumers with cow milk allergies^3^.

Milk adulterations have already been targeted through MALDI-TOF MS analysis, focusing on the analysis of either proteins and peptides^4^ or lipids^5,6^. Profiling of lipids by MALDI MS has been successfully used for the discrimination of coconut, soya and bovine milk^5^. It is a rapid and inexpensive method for the detection of cow milk as an additive to coconut and soya milk^7,8^ that could lead to life-threatening consequences^9^.

However, lipid profiles can often be very similar amongst related species such as ruminants. More importantly, compared to proteins, lipids are relatively small molecules without a species-specific sequence, which can be utilized to identify underlying species.

Previously, the distinction of different mammalian species has been successfully achieved by profiling proteinaceous milk components with a MALDI-TOF MS platform^4^. The reported platform was capable to profile proteins and peptides, although in separate analyses with fundamentally different milk sample preparations, calibration routines, and for peptides, additional instrument settings. In addition, all milk samples apart from skimmed milk were defatted by a centrifugation step of 30 min prior to MALDI sample preparation and molecular cut-off filtering with a purification step was necessary in the case of peptides.

In this work, we propose a simple and fast untargeted MS profiling method by utilizing liquid AP-MALDI^10,11^, reducing sample preparation and introduction times as well as the need to find ‘sweet spots’ for optimal analyte ion signals. Liquid MALDI samples provide robust and stable ion signal yields^12^ of metabolites, lipids and multiply charged proteinaceous analytes^10^ in the same analysis/spectrum. This analytical feature together with the possibility to sequence proteins of interest directly from the same MALDI sample by rapidly switching to CID (and potentially ECD/ETD) MS/MS is currently unique for MALDI MS profiling analysis^13^. The combination with a purpose-built machine learning tool based on Linear Discriminant Analysis (LDA) allows for the real-time recognition of milk from different species and the detection of 5% of cow milk in goat milk at high accuracy.

## Results

One of the main objectives of this study was the development of a user-friendly MS-based method capable of determining milk adulteration to levels as low as 5% of cow milk added to goat milk.

**Figure 1:**
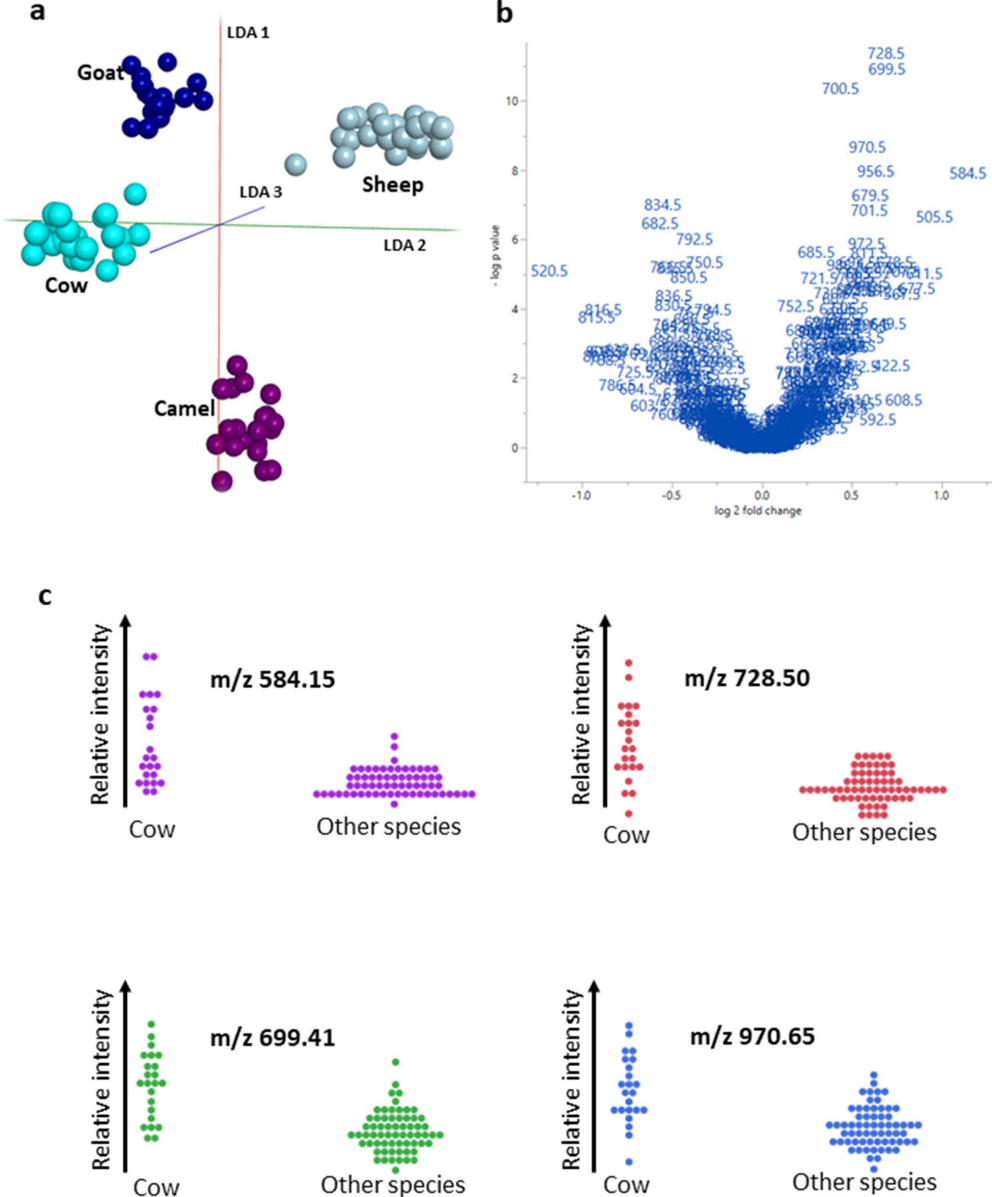
(**a**) Linear Discriminant Analysis (LDA) plot obtained with Abstract Model Builder (AMX) from the MS profile data of sample sets of four different mammals, using the *m/z* range from 400 to 1000. (**b**) Volcano plot displaying the m/z values of ions linked to cow milk classification. (**c**) Graphical representation of some of the most significant differences in the relative signal intensities for lipid ions that are highly relevant for cow milk classification. Based on their m/z values the peaks at m/z 699.41 and m/z 728.50 can be putatively assigned to the [M + H]^+^ ion species of glycerophosphoinositol PI 24:0 and glycerophosphoethanolamine PE 35:3 or glycerophosphocholine PC 32:3 , respectively.

The newly devised protocol for this purpose (see Methods section for details) was first used to acquire MS profiles of unadulterated pure milk with data acquisition times of 10 s per sample from a set of four different mammals (camel, cow, goat and sheep). Figure 1 displays the LDA plot based on the lipid profiles obtained from 20 technical replicates using the AMX program. The model obtained from LDA was then used to classify 64 unknown samples, achieving a 100% classification accuracy for these samples, although the software’s built-in ‘leave 20% out’ cross correlation testing of the data from the samples used for LDA model building resulted in an accuracy of only 98%.

**Figure 2:**
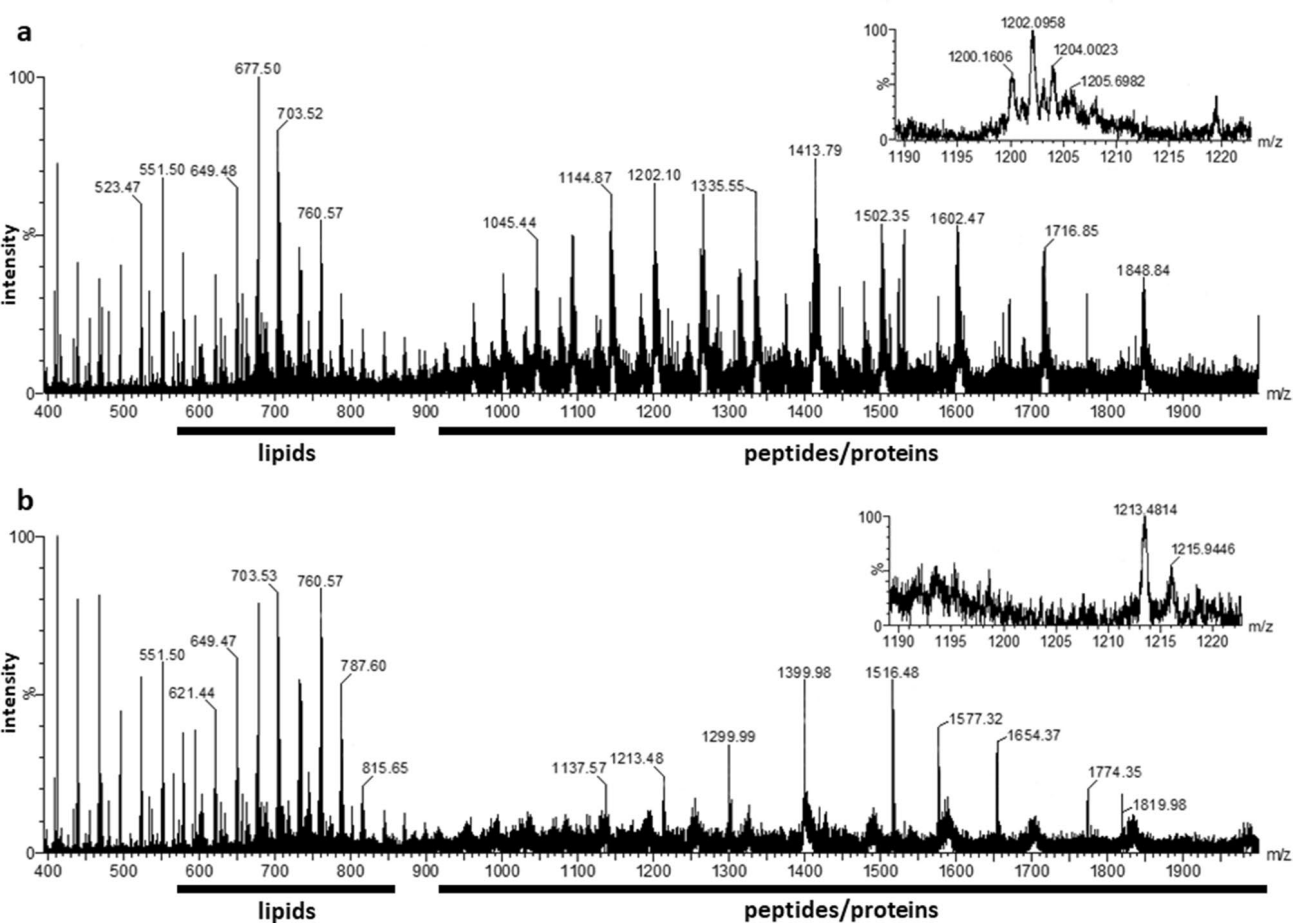
Liquid AP-MALDI mass spectra of cow milk (**a**) and goat milk (**b**), each acquired over 5 min.

For detecting small amounts of cow milk in goat milk, a wider *m/z* range was used to take into account additional milk components such as proteins and their fragments that offer species-specific sequence information that lipids do not provide. Therefore, a new milk preparation method was developed capable of the detection of both lipids and larger hydrophilic biomolecules such as larger peptides and proteins/proteoforms. In short, a conventional TCA precipitation preparation was applied which also retained a sufficient number of lipids^13^. Figure 2 shows two liquid AP-MALDI mass spectra that were acquired with this new milk sample preparation, displaying singly charged lipids up to *m/z* 900 and multiply charged peptides and proteins above *m/z* 800 for cow milk and goat milk, respectively. In particular, the region of the multiply charged proteinaceous ions reveals substantial visible differences between the two species.

**Figure 3:**
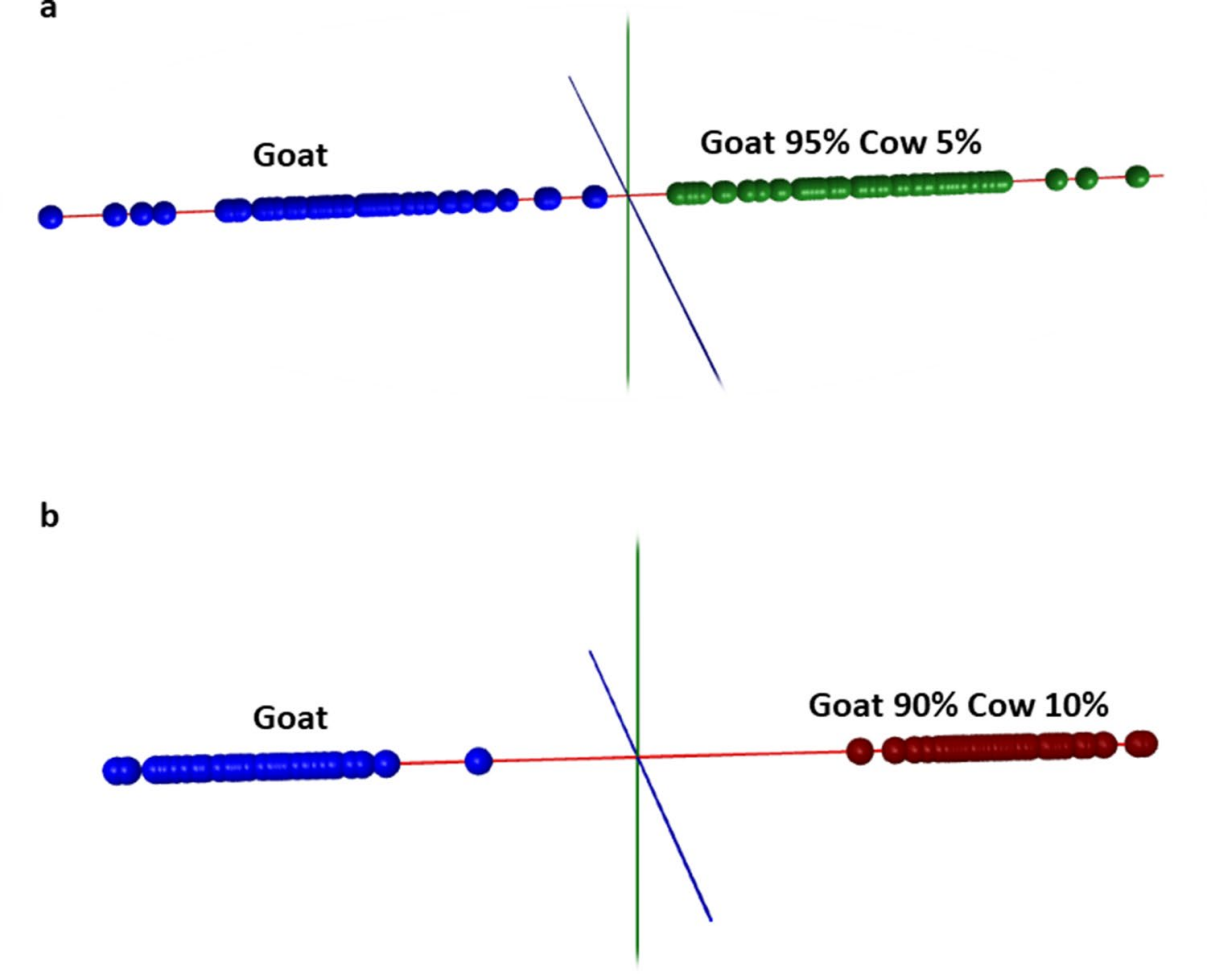
Linear Discriminant Analysis (LDA) obtained with Abstract Model Builder (AMX) showing full separation of all 100% goat milk samples (n = 50) from all goat milk samples adulterated with 5% cow milk (n = 54) (**a**) and from all goat milk samples adulterated with 10% cow milk (n = 48) (**b**).

In order to detect goat milk adulteration by adding cow milk at low levels, the Abstract Model Builder program was trained using 100% goat milk (50 spectra) and goat/cow milk mixtures with 5% cow milk (54 spectra) and 10% cow milk (48 spectra), respectively, using two biological replicates, two mixture replicates and four sample preparation replicates for each sample group. The LDA plot obtained from the analysis of goat milk and goat milk with the 5% cow milk is shown in Fig. 3a, while the LDA plot for the same analysis but with 10% cow milk is depicted in Fig. 3b.

**Figure 4:**
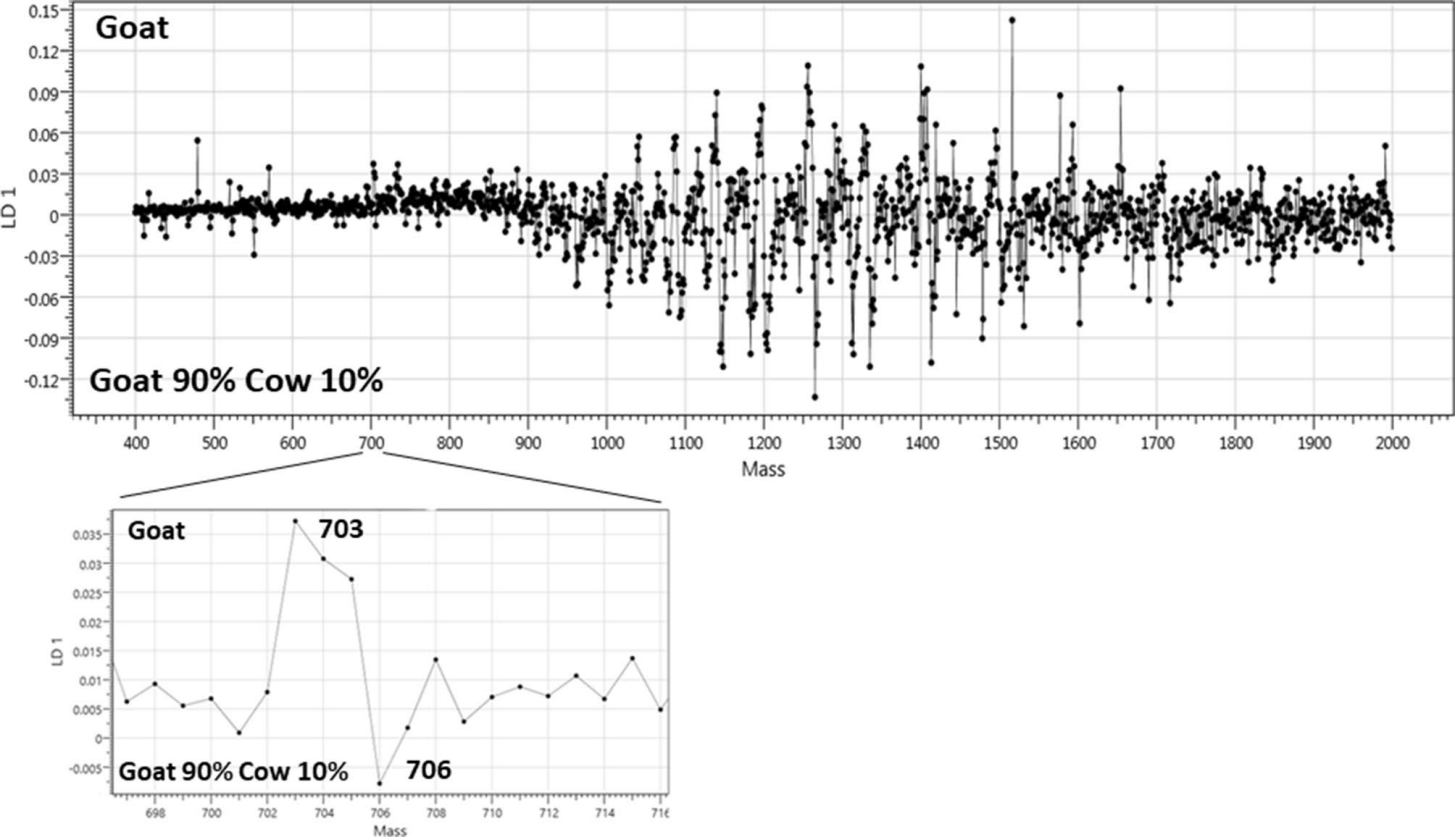
Loading plot of the Linear Discriminant Analysis (LDA) for separating pure goat milk and goat milk adulterated with 10% cow milk.

The mass bins mainly responsible for the separation by LDA can be found in the loading plot shown in Fig. 4. The positive part of the y axis represents a positive correlation between the peak’s intensity and its contribution to classifying the sample as 100% goat milk, while the negative part of the y axis represents a positive correlation between the peak’s intensity and its contribution to classifying the sample as adulterated goat milk.

Mass bin intensities of the most influential peaks in the classification have further been analysed by JMP software. Figure S1 (see Supporting Information) displays the obtained box plots related to the most influential ions found in the LDA separation. The intensity of the ions at *m/z* 1265 (Fig. S1a) and 1335 (Fig. S1b) increases with the concentration of cow milk in each case. Such increase is naturally preferred as the appearance of specific cow-related milk components is a more robust indicator for adulteration than the decrease of goat-specific ion signals such as the signals at *m/z* 1516 and 1256 (see loading plot in Fig. 4). Interestingly, removing the data from the m/z region up to 900 where lipids are detected, caused a decrease of around 5% in the model’s cross-validation accuracy.

The two LDA models obtained for each adulteration case using the m/z region of 400–2000 were then used for the classification of an operator-unknown set of samples consisting of pure goat milk (n = 112) and goat milk adulterated with 5% cow milk (n = 106), and another operator-unknown set of samples consisting of pure goat milk (n = 118) and goat milk adulterated with 10% cow milk (n = 112). The classification results achieved are presented in the confusion matrices shown in Tables 1 and 2.

**Table 1:** Confusion matrix for the detection of 5% cow milk in goat milk using an LDA prediction model based on liquid AP-MALDI MS profiles.

		Condition		
		Positive (5% cow milk in goat milk)	Negative (100% goat milk)	
Test results	Positive (5% cow milk in goat milk)	TP 98	FP 5	Positive predictive value ≈ 95.1%
	Negative (100% goat milk)	FN 8	TN 107	Negative predictive value ≈ 93.0%
		Sensitivity ≈ 92.5%	Specificity ≈ 94.5%	

The ‘Condition’ columns indicate the real nature of the samples analyzed. The rows ‘Test results’ indicate the results obtained by the classification model. Positives are defined as milk samples with 5% cow milk. Negatives are defined as milk samples without cow milk.

*TP* true positives, *FP* false positives, *FN* false negatives, *TN* true negatives.

**Table 2:** Confusion matrix for the detection of 10% cow milk in goat milk using an LDA prediction model based on liquid AP-MALDI MS profiles.

		Condition		
		Positive (10% cow milk in goat milk)	Negative (100% goat milk)	
Test results	Positive (10% cow milk in goat milk)	TP 111	FP 1	Positive predictive value ≈ 99.2%
	Negative (100% goat milk)	FN 1	TN 117	Negative predictive value ≈ 99.1%
		Sensitivity ≈ 99.2%	Specificity ≈ 99.1%	

The ‘Condition’ columns indicate the real nature of the samples analyzed. The rows ‘Test results’ indicate the results obtained by the classification model. Positives are defined as milk samples with 10% cow milk. Negatives are defined as milk samples without cow milk.

*TP* true positives, *FP* false positives, *FN* false negatives, *TN* true negatives.

As seen in Table 1, the addition of cow milk to a final concentration of 5% in goat milk could be detected with a sensitivity of 92.5% and a specificity of 94.5%. The addition of cow milk to a final concentration of 10% in goat milk could be detected with a sensitivity of 99.2% and a specificity of 99.1% (Table 2).

## Discussion

The detection and analysis of adulteration in milk is often undertaken by techniques based on PCR or enzyme-linked immunosorbent assay (ELISA)^14–17^. These techniques can be very fast and sensitive but are dependent on the ligand and substrate interaction. PCR is dependent on the availability or the synthesis of primers^18^, and ELISA is dependent on the production of monoclonal antibodies^19^. Thus, these techniques are not easily adaptable to analyses other than the ones for which they have specifically been designed and optimized. For example, it is virtually impossible to use an ELISA method developed for the detection of cow milk for the specific detection of sheep milk without changing the antibody. Similarly, in most cases new primers are needed to detect other species by PCR.

However, milk analyses can often benefit from the relatively large differences in fatty acid (FA) composition between species. As previously documented, ovine milk has the highest lipid content with the highest amount of monounsaturated FAs and odd- and branched-chain FAs compared to bovine milk, which has been found to be richer in saturated FAs, and milk from mares and donkeys, which have the comparatively highest amount of polyunsaturated FAs^20^. These differences among species in FA composition already represent a good source of information for a lipids-based classification using mass spectral profiles. Some ion signals in the *m/z* range of the lipids also contribute to the classification of the milk from the four different ruminant species and the separation of the pure goat milk from the adulterated goat milk in the presented study. As previously demonstrated by Calvano and colleagues^6^, a lipid ion species detected at *m/z* 703.5, which is also evident in the loading plot of Fig. 4, is more abundant in goat than cow milk and can be effectively used in combination with other lipid ion signals for separating goat from cow milk.

On the other hand, as described in the introduction section, liquid AP-MALDI MS uniquely provides several advantages compared to previous (conventional) MALDI MS profiling work of milk performed on axial TOF mass analyzers. As reported in one of the latest such studies, combined peptide and protein profiling analysis required substantial sample preparation and various modes of MS data acquisition and lacked the detection of lipid profiles^4^. However, exploiting all milk components such as metabolites, lipids and proteins allows the development of an even more powerful analytical tool that can also be better adapted to other applications. In this study, speciation of a few different types of ruminants was easily achieved by simple lipid profiling using liquid AP-MALDI MS while food adulteration was detected by involving the proteoform profiles and/or using tandem mass spectrometry (MS/MS)^13^. In previous work^13^, it was already shown that the individual multiply charged proteinaceous ion species can also be exploited for direct amino acid sequencing by a top-down approach, which is possible by using liquid AP-MALDI on high-performing instruments, thus further underlining the strengths of liquid AP-MALDI in speciation and adulteration analysis.

In both examples presented in this study, LDA-based machine learning approaches extremely simplified the computational analysis and can provide classification in real time. These approaches do not require any molecular synthesis or engineering, but a few simple steps to teach the classification models with a training dataset. In the case of detecting milk adulteration by liquid AP-MALDI MS, it is evident that the mass spectral profiles are extremely rich, showing a plethora of diagnostic ions, including lipids and proteinaceous components. This mass spectral information coupled with LDA-based machine-learning prediction models led to the automatic recognition of cow, goat, sheep and camel milk in just 10 s of analysis per unknown sample with an overall 100% classification accuracy. Regarding the application on adulterations, as can be seen in the loading plot of Fig. 4, the proteinaceous milk components provide the strongest contribution in distinguishing pure goat milk from adulterated goat milk, demonstrating the substantial improvement that can be obtained by utilizing the protein part of milk samples.

In the second application example, in 30 s, this newly developed method allowed the detection of 5% cow milk in goat milk with a positive predictive value of more than 95% (Table 1). The detection of 10% cow milk in goat milk was possible with a sensitivity and specificity as well as positive predictive value of more than 99% (Table 2). As before, the inclusion of multiply charged proteinaceous milk components to the analysis increases the accuracy of the classification by 10%. The ion signal of individual bovine-specific proteinaceous ions (e.g. at *m/z* 1265 and 1335), as detected by liquid AP-MALDI MS and shown in Figure S1, increases with the concentration of cow milk, demonstrating that these peaks are clearly correlated to cow milk addition. The inset in Fig. 2a magnifies the *m/z* region of 1190–1220 with strong signals of highly charged analyte ions. These ions can be attributed to the proteoforms of β-casein with 20 positive charges^13^, which are not present in the mass spectrum of goat milk (see inset of Fig. 2b).

In conclusion, we have provided two examples for the application of liquid AP-MALDI MS profiling coupled with machine-learning and multivariate data analysis in milk sample assessment. Specifically, speciation analysis and the detection of food adulteration were possible using a simple one-pot sample preparation of the crude biofluid milk. This new method resulted in being fast, robust and highly accurate as it produces molecular fingerprints consisting of lipids and proteins with stable ion yields and low sample consumption.

The outcome of these findings demonstrates the potential impact of modern MALDI MS profiling on future analytical strategies for food analysis towards single versatile platforms capable of multiplexing and applicable to a wide range of analyses of different food biomarkers and for different food assessment purposes.

Data supporting the results reported in this paper are openly available from the University of Reading Research Data Archive at 10.17864/1947.232.

## Methods

### Samples and sample preparation

All analyses were performed on fresh, full-fat pasteurized milk. Cow and goat milk was purchased from Waitrose local stores, sheep milk was purchased from Sheep Milk Company Ltd (Preston, UK) and camel milk was purchased from Desert Farms Ltd (London, UK). For each species, two different batches of milk were obtained within 30 days and used as biological replicates. Cow, goat, sheep and camel milk were aliquoted and frozen at − 80 °C immediately after purchase. Aliquots of mixtures of cow-goat milk (0, 5 and 10% of cow milk in goat milk) were also made before freezing all aliquots. All chemicals were purchased from Sigma-Aldrich and used without further purification unless otherwise specified.

For the speciation analysis based on lipids, 50 µl of milk was mixed with 450 µl of hexane/isopropanol (3:2; v/v) and vortexed for 5 s. No centrifugation was required, and the supernatant (lipids fraction) was ready for analysis.

For the adulteration analysis, i.e. the detection of cow milk in goat milk, spectral profiling was performed on both lipids and proteins. For the sample preparation, 50 µl of milk was subjected to a precipitation step carried out with 250 µl of 5% trichloroacetic acid (TCA; w/v). Samples were then centrifuged for 2 min at 12,300 g. The supernatant was discarded and the pellet was re-suspended in a solution of water/acetonitrile/isopropanol (1:1:1; v/v/v), pipetting the solution 10 times within the same vial. After a sonication step of 60 s the analyte solution was directly analyzed or stored at − 20 °C prior to analysis.

All analyte solutions were analysed by liquid AP-MALDI MS^10,21,22^. Briefly, liquid MALDI samples were prepared using α-cyano-4-hydroxycinnamic acid (CHCA) as the MALDI matrix chromophore. CHCA was dissolved in acetonitrile/water (70:30; v/v) by 2-min sonication to a final concentration of 30 mg/ml. Once CHCA was completely dissolved, this solution was diluted 10:7 (v/v) with ethylene glycol, vortexed for 5 s and sonicated for 1 min. This solution represents the liquid support matrix (LSM).

Immediately before MS analysis, 0.7 µl of analyte solution was mixed with 0.7 µl of LSM directly on the MALDI sample plate.

### Mass spectrometry analysis

MS analysis was performed on a hybrid quadrupole-time-of-flight mass spectrometer (Synapt G2-Si; Waters Corporation, Wilmslow, UK) equipped with an ion mobility cell for travelling wave ion mobility spectrometry (TWIMS). The ionization source was an in-house developed and built AP-MALDI source as previously described^11^. Briefly, the ion source consists of a heated ion transfer tube for improved MALDI plume desolvation conditions by applying a temperature of approximately 250–350 °C. In the instrument data acquisition software, the ion block temperature was set to 80 °C and the cone voltage to 40 V. The instrument was employed in sensitivity and ion mobility-TOF mode. The ion mobility wave velocity was set at 650 m/s with a 40 V height and a nitrogen flow of 90 ml/min. Complete IM-MS parameters can be found in the ‘_extern.inf’ file within each raw data folder. These folders are available in the University of Reading’s Research Data Archive as detailed below (see Data availability). The scan time was set to 0.1 s for rapid lipid analysis and to 1 s for the combined analysis of lipids, peptides and proteins. Positive ions were recorded over an *m/z* range of 100–2000.

The samples were irradiated with a pulsed nitrogen laser (337 nm; 3 ns; MNL 103 LD; LTB Lasertechnik GmbH, Berlin, Germany) with a pulse energy of 20 µJ per shot focused to a diameter of approximately 100–150 mm and a pulse repetition rate of 20 Hz.

External TOF calibration was performed by AP‐LDI MS as previously described^23^ using sodium iodide and an acquisition time of 3 min with an *m/z* range of 100–2000 using Intellistart (MassLynx, Waters). MS data acquisition of MALDI samples was automated using Waters Research Enabled Software (WREnS).

Data acquisition time was 10 s per sample for the lipid-only analysis and 30 s per sample for the analysis of lipids, peptides and proteins. The sum of around 100 scans per sample was considered for the creation of the multivariate model for the analysis of lipids while it was around 30 scans per sample for the combined analysis of lipids, peptides and proteins.

### Data processing

For each sample, the final mass spectrum used was obtained by summing all scans for that sample discarding the ion mobility retention time information. Raw datasets were then analyzed with Abstract Model Builder (AMX; version 1.0.1563.0; Waters).

In general, the classification models described in this study were built using a training set and cross-validated with the AMX tool ‘leave 20% out’. Subsequently, each model was tested using the data from an unknown test sample set. These classification test results were manually validated. For both lipid-only analysis and the combined lipid/peptide/protein analysis, one third of the milk samples was used to build the classification model. Test milk samples that were not used to build the model were then analyzed with the AMX Recognition tool of AMX by loading the created Linear Discriminant Analysis (LDA) model. For lipid analysis applied to speciation, an LDA model was built based on the *m/z* range of 400–1000. For the combined lipid/peptide/protein analysis, a model was built using the *m/z* range of 400–2000.

Models were build using the following parameters: binning intensities every 1 Thomson; advanced binning mode; application of the AMX built-in normalization tool. The data matrices obtaining the normalized intensities of each mass bin, together with the data acquisition (chromatogram) times and the experimental group of each sample have been included as .csv files, which are available in the University of Reading’s Research Data Archive as detailed below (see Data availability). Using these files it is possible to map each spectrum of every sample in the chromatogram of the .raw files.

The relative intensities of the ions mainly responsible for the classification were subsequently analyzed with JMP software (version 14; SAS Institute Inc., Marlow, UK).

## Supplementary Information

Supplementary Information

## Data Availability

Data supporting the results reported in this paper are openly available from the University of Reading Research Data Archive at 10.17864/1947.232.

## Abbreviations

AP: Atmospheric pressure
ELISA: Enzyme-linked immunosorbent assay
ESI: Electrospray ionization
FA: Fatty acid
HPLC: High-performance liquid chromatography
LDA: Linear discriminant analysis
LDI: Laser desorption/ionization
LOD: Limit of detection
LSM: Liquid support matrix
MALDI: Matrix-assisted laser desorption/ionization
MS: Mass spectrometry
PCR: Polymerase chain reaction
TCA: Trichloroacetic acid
WREnS: Waters research enabled software
