# Supplementary material for: Speciation and milk adulteration analysis by rapid ambient liquid MALDI mass spectrometry profiling using machine learning
Source: Sci Rep. 2021 Feb 8;11:3305. doi: 10.1038/s41598-021-82846-5 (PMC7870811; doi:10.1038/s41598-021-82846-5)
Supplement: Supplementary file 1 — Supplementary Information [file 41598_2021_82846_MOESM1_ESM.pdf]

Supporting Information to 'Speciation and milk adulteration analysis by rapid ambient liquid MALDI mass spectrometry profiling using machine learning'

*Cristian Piras,[1] Oliver J. Hale[1†], Chris Reynolds[2], A K (Barney) Jones[2], Nick Taylor[3], Mike Morris[4], Rainer Cramer[1]**

[1] Department of Chemistry, University of Reading, Whiteknights, Reading, RG6 6AD, UK

[2] School of Agriculture, Policy and Development, PO Box 237, Earley Gate, Reading RG6 6AR Berkshire UK

[3] Veterinary Epidemiology and Economics Research Unit (VEERU) & PAN Livestock Services Ltd., University of Reading, School of Agriculture, Policy and Development, Reading RG6 6AR, UK

[4] Waters Corporation, Stamford Avenue, Wilmslow SK9 4AX, U.K

[†] Present Address: School of Biosciences, University of Birmingham, Edgbaston, Birmingham, B15 2TT, UK

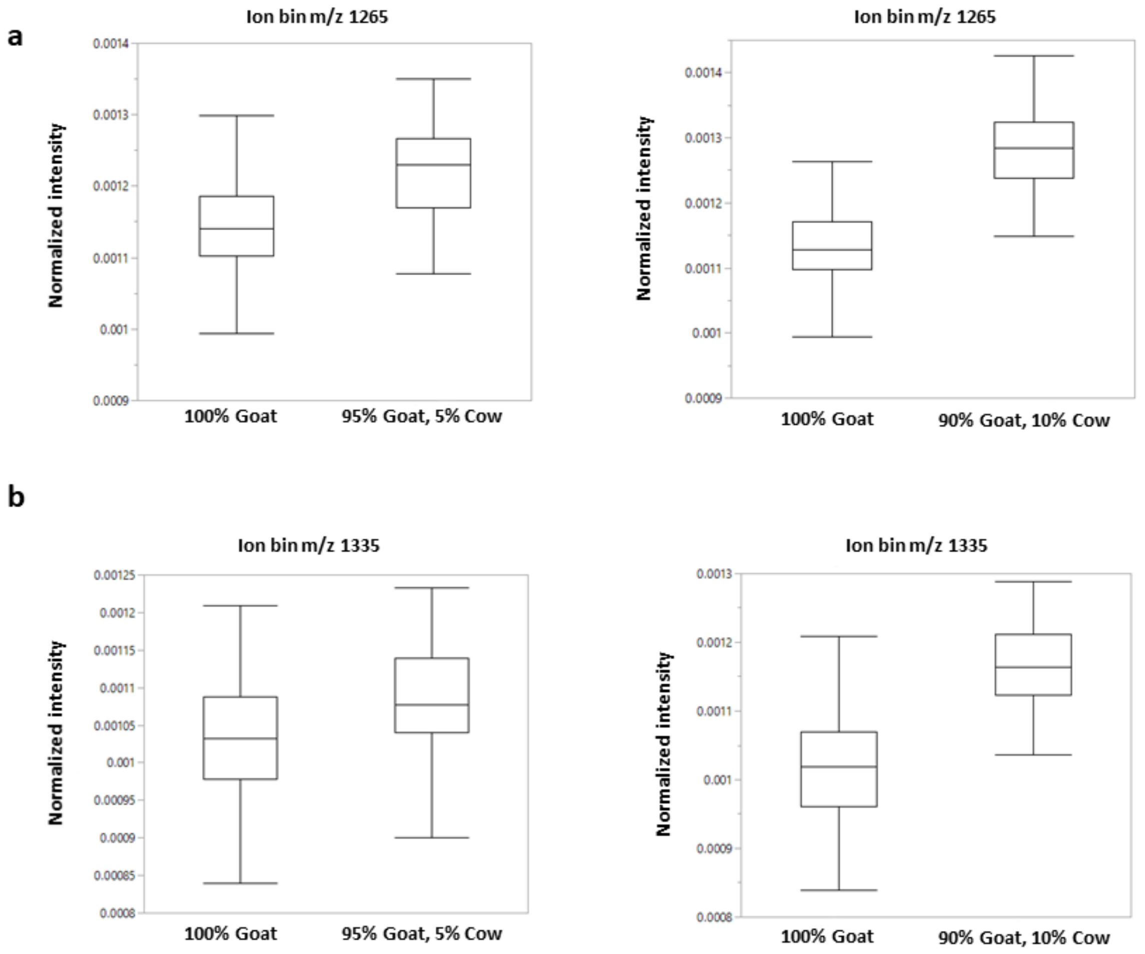

Figure S1. SAS jmp outlier box plots of the mass bin ion intensities for the milk profile peaks at m/z 1265 and 1335. The *t*-test comparison between the 100% goat and the 95% goat (5% cow) milk showed significant differences for both mass bins *m/z* 1265 ($p = 4.2*10^{-8}$) and 1335 ($p = 0.000415$). The p-values for the same mass bins for the 10% cow contamination are even lower.
